# Supplementary material for: Molecular Fingerprint of Endocannabinoid Signaling in the Developing Paraventricular Nucleus of the Hypothalamus as Revealed by Single-Cell RNA-Seq and In Situ Hybridization
Source: Cells. 2025 May 27;14(11):788. doi: 10.3390/cells14110788 (PMC12153593; doi:10.3390/cells14110788)
Supplement: Supplementary file 1 [file cells-14-00788-s001.zip › Supporting Information R1 FINAL.pdf]

Supplementary Information for

Molecular fingerprint of endocannabinoid signaling in the developing paraventricular nucleus of the hypothalamus as revealed by single-cell RNA-seq and *in situ* hybridization

Evgenii Tretiakov, Zsófia Hevesi, Csenge Böröczky, Alán Alpár,
Tibor Harkany, and Erik Keimpema

Containing:

Abbreviations
Supplementary Table 1
Supplementary Figures 1-3
Supplementary References

Abbreviations

2-AG	2-arachidonoylglycerol
3V	Third ventricle
Abhd4/6/12 (ABHD4/6/12)	α/β-hydrolase domain containing 4/6/12
AEA	Anandamide
AHN	Anterior hypothalamic nucleus
ATP	Adenosine triphosphate
Avp (AVP)	Arginine vasopressin
Cnr1/2 (CB_1R)	Cannabinoid receptor type 1/2
Cox2 (COX2)	Cyclooxygenase-2
Crh (CRH)	Corticotrophin-releasing hormone
Dagla/b (DAGLα/β)	Sn1-specific diacylglycerol lipase alpha/beta
E	Embryonic
Faah (FAAH)	Fatty-acid amide hydrolase
Gde1 (GDE1)	Glycerophosphodiester Phosphodiesterase 1
Gpr55 (GPR55)	G protein-coupled receptor 55
HPA	Hypothalamic-pituitary-adrenal
Lox (LOX)	Lipoxygenase
MS	Medial septum
Mgll (MGLL)	Monoacylglycerol lipase
Napepld (NAPE-PLD)	N-acyl phosphatidylethanolamine phospholipase D
OB	Olfactory bulb
OT	Olfactory tract
P	Postnatal
Ptpn22 (PTPN22)	Protein tyrosine phosphatase non-receptor type 22
PVN	Paraventricular nucleus
SCN	Suprachiasmatic nucleus
Sst (SST)	Somatostatin
Trh (TRH)	Thyrotropin-releasing hormone
Oxt (OXT)	Oxytocin
UMAP	Uniform Manifold Approximation and Projection
UpSet	Visualization of Intersecting Sets

Tables

Supplementary Table S1. Semi-quantitative *in situ* hybridization analysis of the 2-AG signaling cassette throughout developmental stages. Plusses indicate expression amounts, *n.d.* stands for "not detected".

Age	Gene	*Cnr1*	*Dagla*	*Daglb*	*Mgll*
E15	*Trh*	+++	*n.d.*	+	*n.d.*
	Crh	++++	*n.d.*	+	*n.d.*
	Oxt	++	*n.d.*	+	*n.d.*
P10	*Trh*	+++	*n.d.*	+	*n.d.*
	Oxt	++	*n.d.*	+	*n.d.*
P21	*Trh*	+++	++	+	+
	Crh	++++	++		+
	Oxt	++	+		+

Figures

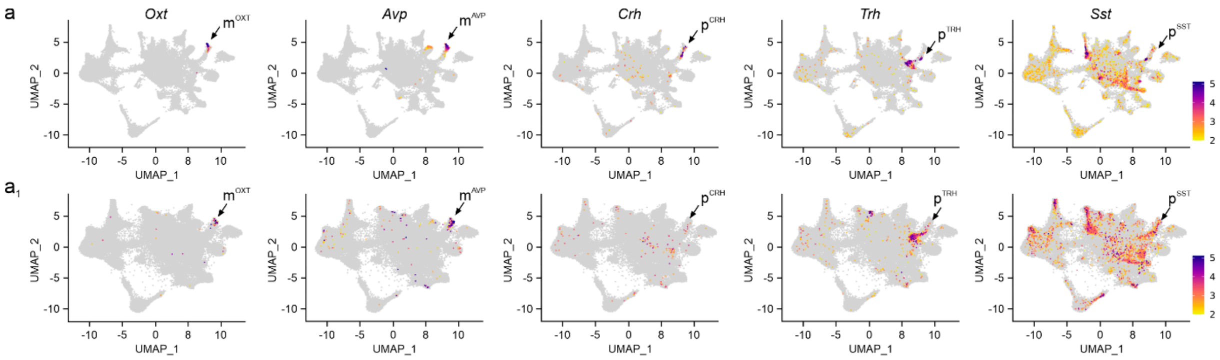

Supplementary Figure S1. Comparison of neuronal clusters for select neuropeptides within the hypothalamus. (**a**) Neuropeptide analysis from our mixed developmental hypothalamic dataset[1] reveals distinct groups of magnocellular (m^{OXT}, m^{AVP}) and parvocellular (p^{CRH}, p^{TRH}, p^{SST}) cells of the PVN on UMAP plots (*arrows*). (**a_1**) Re-analysis of a previously published developmental hypothalamic RNA-seq study confirms our clustering[2], with our dataset more clearly resolving p^{CRH} and p^{TRH} populations. Relative expression is color-coded on the right for each gene analyzed.

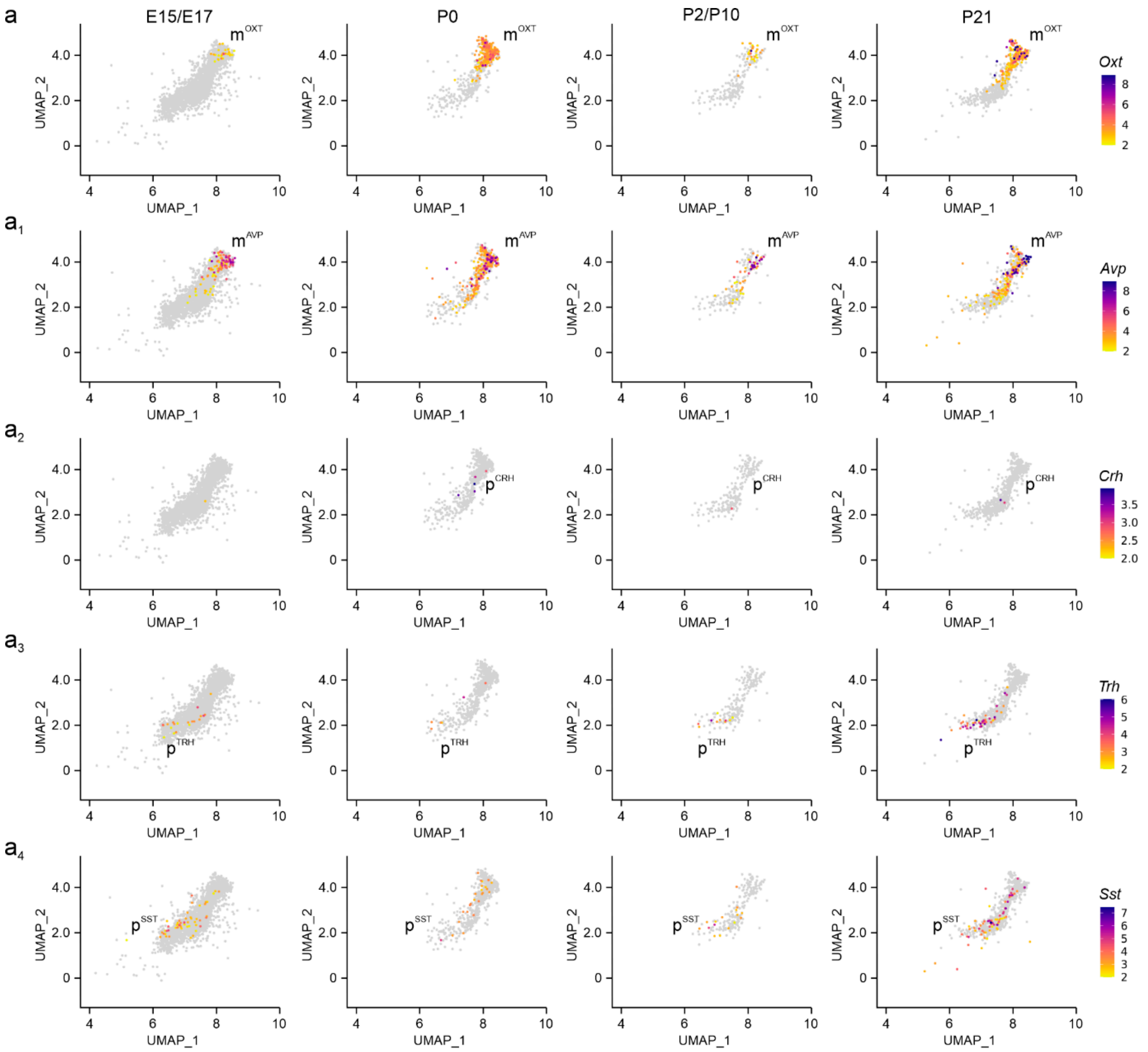

Supplementary Figure S2. Comparison of neuronal clusters for select neuropeptides within the PVN. (a-a$_4$) Re-analysis of a previously published developmental hypothalamic RNA-seq study confirms our clustering[2] of neurons in the PVN. Relative expression is color-coded on the right for each gene analyzed.

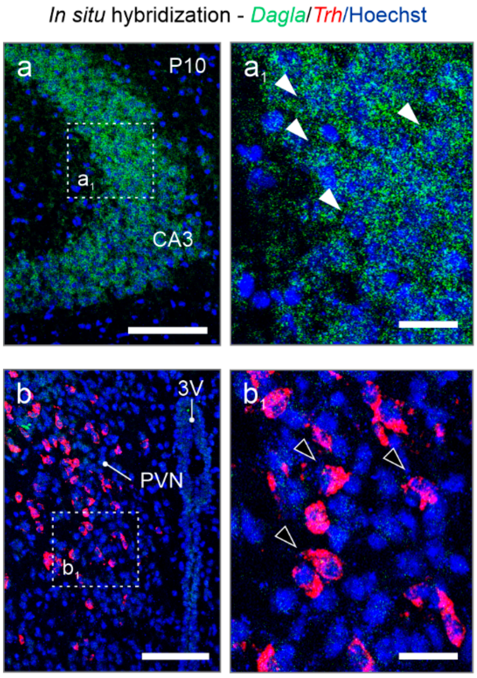

Supplementary Figure S3. *Dagla* in situ probe validation. (**a,a_1**) In situ hybrization for *Dagla* reveals a strong labeling in the hippocampal CA3 region (*arrowheads*) at postnatal day 10. (**b,b_1**) On the same section, there was limited *Dagla* labeling detected in the PVN (*open arrowheads*). Abbreviations: 3V, third ventricle; CA3, cornu ammonis 3; PVN, paraventricular nucleus. *Scalebars* = 300 µm (a,b), 20 µm (a_1,b_2).

References

1. Romanov, R. A. *et al.* Molecular design of hypothalamus development. *Nature* 1–7 (2020) doi:10.1038/s41586-020-2266-0.

2. Kim, D. W. *et al.* The cellular and molecular landscape of hypothalamic patterning and differentiation from embryonic to late postnatal development. *Nat Commun* **11**, 4360 (2020).
